# A new species of the genus *Parachemmis* Chickering, 1937 from Colombia (Araneae, Corinnidae, Corinninae)

**DOI:** 10.3897/zookeys.679.12421

**Published:** 2017-06-08

**Authors:** Leonel Martinez, Eduardo Villarreal, Neis Martinez

**Affiliations:** 1 Grupo de Investigación ECOSIN, Semillero de Investigación Artrópodos e Insectos Neoptera del Caribe Colombiano, Departamento de Biología, Universidad del Atlántico, Barranquilla, Colombia; 2 Grupo de Investigación Biodiversidad del Caribe colombiano. Programa de Biología, Universidad del Atlántico, Barranquilla

**Keywords:** Dionycha, distribution, Neotropical Region, Sierra Nevada de Santa Marta, taxonomy

## Abstract

The spider genus *Parachemmis* Chickering, 1937 (Araneae: Corinnidae: Corinninae) is reported from Colombia for the first time. *Parachemmis julioblancoi* **sp. n.** Martinez-G & Villarreal is described and illustrated from the Sierra Nevada de Santa Marta, Magdalena department. The exclusive morphology of the short and apically truncated retrolateral tibial apophysis and club-like tegular laminar process of the male palp indicates that the specimens described herein belong to a new species of *Parachemmis*. A map of the distribution of species in the genus is included.

## Introduction

The family Corinnidae Karsch, 1880 currently includes 754 species in 67 genera (World Spider Catalog 2017) in two subfamilies (Ramírez 2014). The subfamily Corinninae includes 17 genera, characterized by two synapomorphies: the male palpal reservoir primarily coiled and with a sclerotized distal sector, this last is share too with some Castianeiriniae (Platnick and Baptista 1995, Bonaldo 2000, Ramírez 2014). The genus *Parachemmis* was proposed by Chickering (1937) with *Parachemmis fuscus* Chickering, 1937 as type species, and placed in the subfamily Micariinae (Clubionidae). Posteriorly Reiskind (1969) considered *Parachemmis* as a Liocraninae by the presence of four to six pairs of long ventral spines on the tibiae of the first pair of legs and setae simply on abdomen, however, Bonaldo and Brescovit (1994) examined this characters and transferred *Parachemmis* to the subfamily Corinninae, because several pairs of ventral spines on anterior tibiae are common among Corinninae and the photograph of the *Parachemmis hassleri* (Gertsch 1942) reveal feathery setae on abdomen and legs (Bonaldo and Brescovit 1994). Currently this genus includes three valid species, distributed in Brazil, Guyana, and Panamá. Nevertheless, these numbers are highly conservative, because in countries considered megadiverse as Colombia, only has reported 12 genera and 25 species for the family Corinnidae (World Spider Catalog 2017; William Galvis pers. comm.). Therefore, it is expected that with greater effort and review of material deposited in Natural History Museums our understanding of this diversity will increase considerably.

The species of the genus *Parachemmis* can be recognized by having serrula in the lateral edge of the endites, subovate carapace, abdominal traqueal tubercule, male palp with retrolateral tibial apophysis (RTA) entire and by the presence of an articulated ventral tibial apophysis (VTA), as well as by the presence of a prolateral laminar process (PLP) arising from the tegulum. In females, the epigyne have two copulatory openings, the copulatory ducts are large and the secondary spermathecae are poorly-developed. On the other hand, specimens of the genus also have anterior eye row strongly procurved, posterior eye row lesser procurved, and anterior median eyes clearly larger than the others, in addition to having the sternum with two external anterolateral excavations; the latter is also characteristic of the genera *Stethorrhagus* Simon, 1896 and *Tupirinna* Bonaldo, 2000 (Bonaldo 2000).

In this paper, both sexes of a new species of *Parachemmis* Chickering, 1937 from Colombia is described and illustrated: *P. julioblancoi* sp. n. from the Sierra Nevada de Santa Marta (SNSM), Magdalena, Colombia. Finally, a map of the distribution of the genus is included.

## Material and methods

The specimens examined are deposited in the Arachnological Collection of the Instituto de Ciencias Naturales of the Universidad Nacional de Colombia (ICN-Ar, Eduardo Flórez), Bogotá. The multifocal photographs of the copulatory structures and the measurements of the specimens were taken with a Leica MC–120 HD digital camera attached to a Leica S8AP0A stereomicroscope, the photographs were united by the image stacking software Leica Application Suite version 4.1.0. The illustrations of the palp and epigyne were made with a light camera attached to a Leica M125 stereomicroscope and the software Inkscape version 0.91. Platnick and Shadab (1975) is used as model for describing leg spination (with minor changes). For visualization of female genitalia, the epigynal plate was dissected and cleared in KOH solution 10% concentration, following the guideline proposed by Platnick et al. (1999).

Abbreviations used in the text and figures are:

**AER** anterior eye row

**ALE** anterior lateral eye

**AME** anterior median eye

**c** conductor

**cd** copulatory duct

**co** copulatory opening

**d** dorsal

**e** embolus

**fd** fertilization duct

**m** meters above mean sea level

**p** prolateral

**PER** posterior eye row

**PLE** posterior lateral eye

**PLP** prolateral laminar process

**PME** posterior median eye

**po** epigynal pocket

**pr** proximal

**r** retrolateral

**RTA** retrolateral tibial apophysis

**sp** spermathecal

**Spe** spermophore

**ST I** primary spermatheca

**ST II** secondary spermatheca

**v** ventral

**VTA** ventral tibial apophysis

The map was prepared in the Geographic Information System QGIS “Las Palmas” (version 2.18.0, http://www.qgis.org/es/site/). The measurements are given in millimeters.

## Taxonomy

### Parachemmis

Taxon classificationAnimaliaAraneaeCorinnidae

Chickering, 1937

Parachemmis Chickering, 1937:38 (type species: Parachemmis fuscus Chickering, 1937)

#### Diagnosis and description.

See Bonaldo 2000: 126.

### Parachemmis julioblancoi

Taxon classificationAnimaliaAraneaeCorinnidae

Martinez-G & Villarreal sp. n.

http://zoobank.org/EE03D67B-E20A-4DCB-98F3-4911195437DE

Figs 1 , 2 , 3

#### Holotype.

Male in ICN-Ar, from Colombia, Magdalena, Sierra Nevada de Santa Marta, San Pedro de la Sierra, 2104 m, 10.895138°N, 73.999611°W, 28 Mar.2017, L. Martínez (ICN-Ar 8420). **Paratypes.** 1♀, same data (ICN-Ar 8421); 2♂ (ICN-Ar 8422- 8423) and 4♀ (ICN-Ar 8424) from same locality; 1 ♂ and 1♀ (ICN-Ar 8327- 8328) same locality. 6 May 2016.

#### Etymology.

The specific epithet is a patronym in honor of Dr. Julio Enrique Blanco (Founder of the Universidad del Atlántico), for his many contributions to art and education in Colombia.

#### Diagnosis.

Males of *P. julioblancoi* sp. n. can be distinguished from all remaining species of the genus by the large, club-like PLP, which extends towards the middle and distal part of the tegulum, wide conductor that ends next to embolus, a short, apically truncated RTA and a wide VTA (Fig. 2A–C), Females of *P. julioblancoi* sp. n. resembles those of *P. fuscus* by having the copulatory openings placed medially and by the non-coiled copulatory ducts, but can be diagnosed by the short, wide and rounded copulatory ducts and very short ST II (Fig. 2D–E).

#### Description.

**Figure 1.**
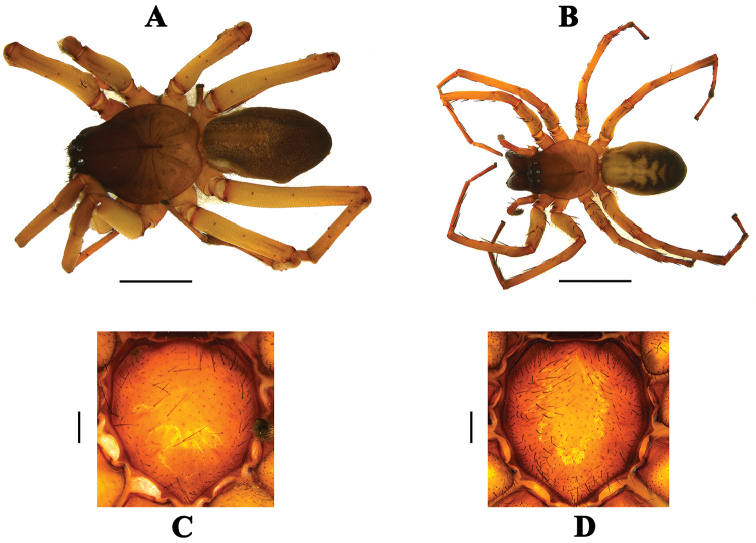
*P. julioblancoi* sp. n., holotype (ICN–Ar 8420), **A** habitus **C** sternum, paratype female (ICN-Ar 8421) **B** habitus **D** sternum. Scale bars 2 mm (**A**) , 5 mm (**B**), 0.3 mm (**C**), 0.5 mm (**D**).

**Male (holotype, ICN–Ar 8327).** Total length: 7.77. Carapace brown, 3.20 long, 2.65 wide, 1.15 high. Eyes AER 1.18 wide, PER 1.32 wide. AME 0.25, PME 0.17, ALE 0.20, PLE 0.21. (Fig. 1A). Sternum brown 1.69 long, 1.65 wide (Fig. 1C). Legs 4123, I-femora 3.10/ patella 0.94/ tibiae 3.40/ Metatarsus 3.01/ tarsus 1.65/total = 12.10; II 3.29/0.99/2.75/2.85/1.63/11.51; III 2.33/0.70/2.32/2.05/1.21/8.61; IV 3.2/1.25/2.67/3.65/1.47/12.24. Chelicerae dark brown, with three promarginal and four retromarginal teeth. Labium brown. Legs yellow (Fig. 1A). Leg macrosetae: femora, I d 1-1-0, p 1 di; II d=I, p 1-1-1; III d 1-1-1, p 1-0-1, r 1-0-1; IV d=III, p 1 di, r 1 di. Tibiae, I v 1–2–2; II v 0–2–2; III v 2-2-2, p 1-0-1, r 1-0-1; IV v=III, p 1-1-0, r1-1-0. Metatarsus, I v 2-0-2; II v 2–0–1; III v 2–2–2, p 1 pr, r 1-1-0; IV v=III, p 1-1-0, r=III. Abdomen Dark gray, with an anterior yellow patch (Fig. 1A); ventrally gray. Spinnerets gray.

**Female. (paratype, ICN–Ar 8328).** Total length: 11.00. Carapace brown with yellowish posterior borders, 4.72 long, 3.84 wide, 1.97 high. Eyes AER 1.35 wide, PER 1.56 wide. AME 0.29, PME 0.24, ALE 0.27, PLE 0.19 (Fig. 1B). Sternum brown 2.42 long, 2.04 wide (Fig. 1D). Legs 4123. I-femora 4.26/ patella 1.46/ tibiae 4.41/ Metatarsus 3.77/ tarsus 1.01/total= 14.91; II 4.32/1.15/4.16/3.41/0.97/14.01; III 4.02/1.16/3.52/3.65/1.36/13.71; IV 5.17/1.49/4.45/5.32/1.61/18.04. Chelicerae dark brown with two promarginal and four retromarginal teeth. Labium brown. Legs yellow (Fig. 1B). Leg macrosetae: femora, I d 1-1-0 , p 0-1-1; II d 1-1-1, p 0-2-1; III d 1 di, p 2-1-1, 1, r 0-1-1; IV d 1-1-1, p 1 di, r 1 di. Tibiae, I-II v 2–2-2; III v 2-2-2-2, p 0-1-1, 0-1-1; IV v 2-2-2, p 1 di, 1-0-1. Metatarsus, I v 2-0-2; II v 2–1–2; III v 2-1-2-2, p 1-1-1, r 1-1-1; IV v 1-1-2-2, p 1-1-0, r 1-0-1. Abdomen dorsal gray, with several anterior yellow patches. (Fig. 1B); ventrally gray. Spinnerets gray.

#### Natural history.

The type material was collected manually, on leaf litter, in a conserved high mountain wet forest ecosystem.

#### Distribution.

Only known from the type locality (Fig. 3).

#### Authors’ contributions.

LM and EV collected, and identified the material. LM was responsible for species description. LM, NM and EV reviewed the literature, drafted the manuscript, and contributed to the critical discussion. EV and LM prepared the images. All authors read and approved the final manuscript.

**Figure 2.**
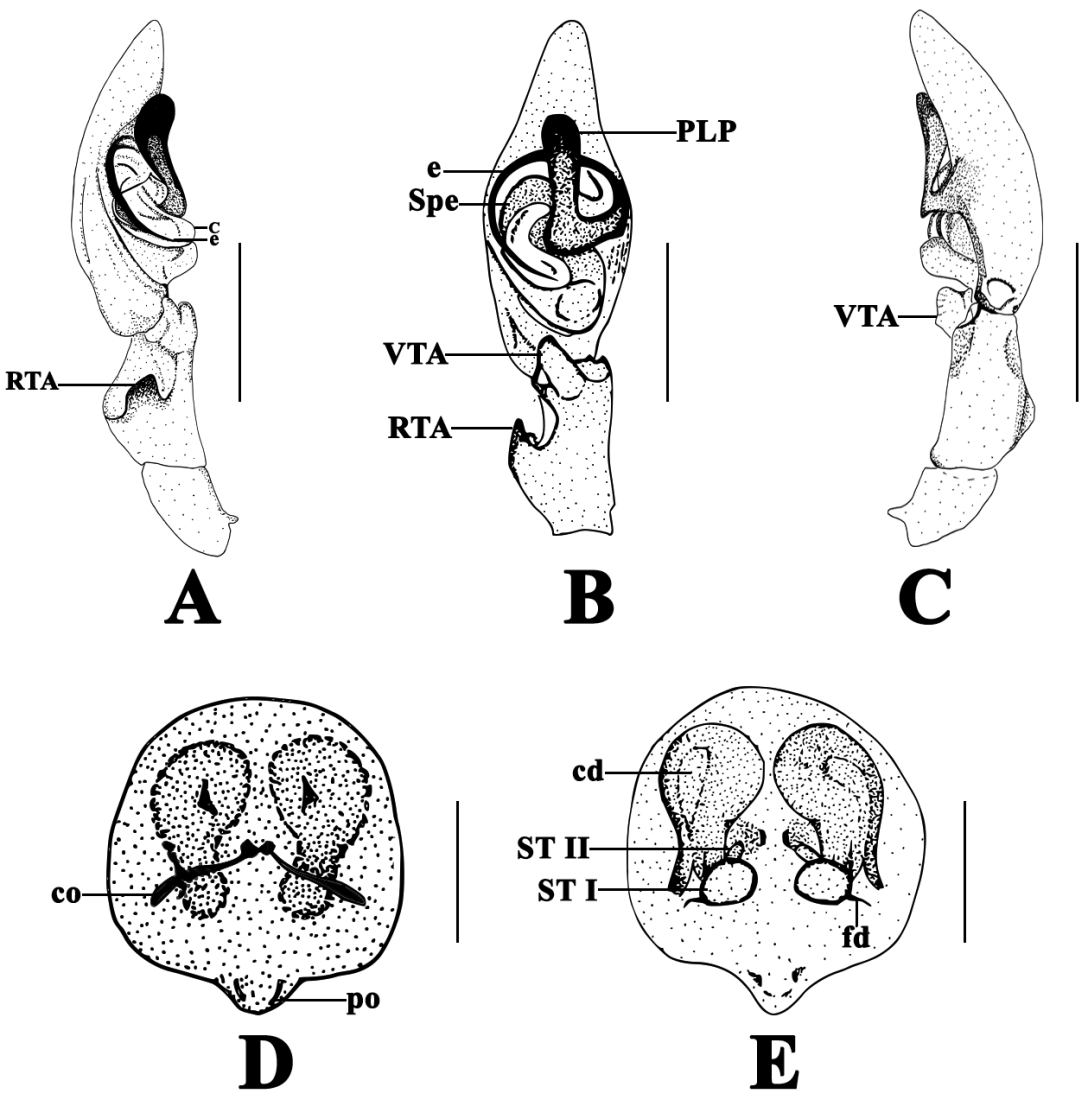
*P. julioblancoi* sp. n., holotype male (ICN–Ar 8420) **A** left male palp, prolateral view **B** same, ventral view **C** same, retrolateral view; paratype female (ICN–Ar 8421) **D** epigyne, dorsal view **E** same, ventral view. Abbreviations: c = conductor; cd = copulatory duct; co = copulatory opening; e = embolus; fd = fertilization duct; PLP = prolateral laminar process; po = epigynal; RTA = retrolateral tibial apophysis; sp = spermathecae; Spe = spermophore; VTA = ventral tibial apophysis. Scale bars 1 mm (**A–C**), 0.5 mm (**D–E**).

**Figure 3.**
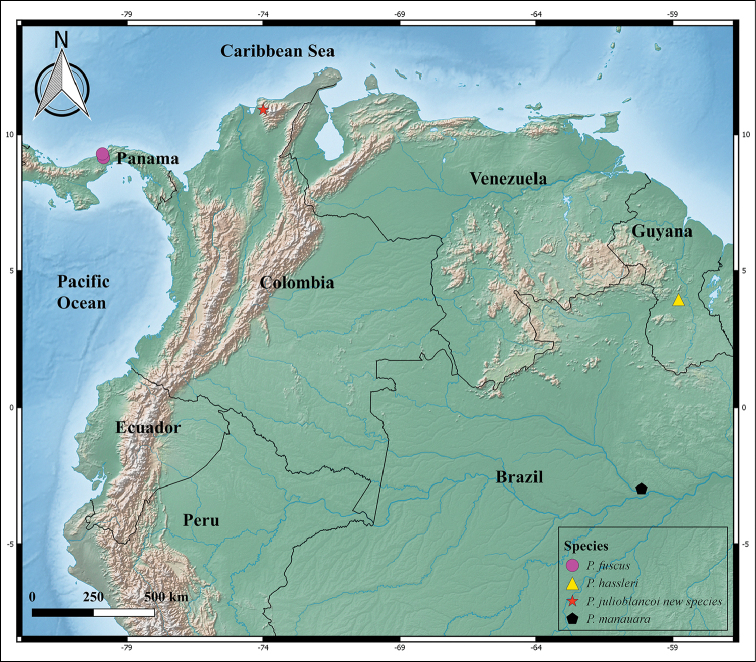
Distribution of the species of the genus *Parachemmis* in South America.

## Supplementary Material

XML Treatment for Parachemmis

XML Treatment for Parachemmis julioblancoi
